# A convolutional neural network-based system to prevent patient misidentification in FDG-PET examinations

**DOI:** 10.1038/s41598-019-43656-y

**Published:** 2019-05-10

**Authors:** Keisuke Kawauchi, Kenji Hirata, Chietsugu Katoh, Seiya Ichikawa, Osamu Manabe, Kentaro Kobayashi, Shiro Watanabe, Sho Furuya, Tohru Shiga

**Affiliations:** 1 Graduate School of Biomedical Science and Engineering, School of Medicine, Hokkaido University, Sapporo, 060-8638 Japan; 2 Department of Nuclear Medicine, Hokkaido University, Sapporo, 060-8638 Japan; 3 Faculty of Health Sciences Biomedical Science and Engineering, Hokkaido University, Sapporo, 060-8638 Japan

**Keywords:** Cancer imaging, Machine learning, Cancer imaging, Biomedical engineering, Computer science

## Abstract

Patient misidentification in imaging examinations has become a serious problem in clinical settings. Such misidentification could be prevented if patient characteristics such as sex, age, and body weight could be predicted based on an image of the patient, with an alert issued when a mismatch between the predicted and actual patient characteristic is detected. Here, we tested a simple convolutional neural network (CNN)-based system that predicts patient sex from FDG PET-CT images. This retrospective study included 6,462 consecutive patients who underwent whole-body FDG PET-CT at our institute. The CNN system was used for classifying these patients by sex. Seventy percent of the randomly selected images were used to train and validate the system; the remaining 30% were used for testing. The training process was repeated five times to calculate the system’s accuracy. When images for the testing were given to the learned CNN model, the sex of 99% of the patients was correctly categorized. We then performed an image-masking simulation to investigate the body parts that are significant for patient classification. The image-masking simulation indicated the pelvic region as the most important feature for classification. Finally, we showed that the system was also able to predict age and body weight. Our findings demonstrate that a CNN-based system would be effective to predict the sex of patients, with or without age and body weight prediction, and thereby prevent patient misidentification in clinical settings.

## Introduction

Patients are sometimes misidentified during imaging examinations. For example, the wrong patient is scanned and/or the wrong images are registered on a picture archiving and communication system (PACS), sometimes leading to severe consequences^1,2^. In some clinical settings, no formal check is conducted to determine whether the obtained images are matched to the correct patient. Various efforts have been made to prevent patient misidentification; for example, many hospitals require that patients wear a wristband with identifying information. This method has significantly reduced the rate of misidentification accidents, but it cannot be applied to outpatients or emergency situations^3^. There remains a demand for a low-cost automated system that can correctly match patients and their images.

Image analyses using a convolutional neural network (CNN), a type of machine-learning algorithm, are gaining attention as an important application of artificial intelligence (AI) to medical imaging^4–7^. CNNs are a class of deep learning techniques that is considered applicable to image analyses because they recognize complex visual patterns in a manner similar to the processes of human perception^8^. Thus, in a study using a CNN, tuberculosis was automatically detected on chest radiographs^9^. In another report, a CNN enabled brain tumor segmentation from magnetic resonance images^10^. Deep learning with a CNN showed high diagnostic performance in the differentiation of liver masses by dynamic contrast agent-enhanced computed tomography^11^. CNNs have also been successfully applied for the detection of lesions and prediction of treatment response by PET^12–14^.

The rate of misidentification accidents could be significantly reduced if AI could predict patient characteristics (e.g., sex, age, and body weight) automatically from a PET-CT or other image alone; this output could then be paired with a system that would issue alerts when data mismatches are detected. We therefore sought to develop and test a CNN system that could predict patient sex, age and body weight from FDG PET-CT images.

## Methods

### Subjects

This retrospective study included 6,462 consecutive patients (3,623 men and 2,839 women; mean age ± SD, 61.6 ± 16.2 years; range 2–92 years) who underwent whole-body FDG PET-CT with either Scanner 1 (n = 5,641) (Biograph 64 PET-CT scanner; Asahi-Siemens Medical Technologies, Tokyo) or Scanner 2 (n = 821) (GEMINI TF64 PET-CT scanner; Philips Japan, Tokyo) at our institute between January 2015 and August 2017. When the same patient was scanned more than once, only the first scan was included. The Institutional Review Board approved the study (#017-0365), waiving the requirement for written informed consent from each patient because of the study’s retrospective nature. We confirmed that all methods were carried out in line with the relevant guidelines and regulations.

### Image acquisition and reconstruction

All clinical PET-CT studies were performed with either Scanner 1 or Scanner 2. All patients fasted for ≥6 hours before the injection of FDG (approx. 4 MBq/kg), and the emission scanning was initiated 60 min post-injection. For Scanner 1, the transaxial and axial fields of view were 68.4 cm and 21.6 cm, respectively. For Scanner 2, the transaxial and axial fields of view were 57.6 cm and 18.0 cm. A 3-min emission scan in 3D mode was performed for each bed position. Attenuation was corrected with X-CT images acquired without contrast media. Images were reconstructed with an iterative method integrated with (Scanner 1) or without (Scanner 2) a point spread function.

The reconstructed images had a matrix size of 168 × 168 with a voxel size of 4.1 × 4.1 × 2.0 mm for Scanner 1, and a matrix size of 144 × 144 with a voxel size of 4.0 × 4.0 × 4.0 mm for Scanner 2. Maximum intensity projection (MIP) images (matrix size 64 × 64) were generated by linear interpolation. In this study, CT images were used only for attenuation correction, not for classification.

### Convolutional neural network (CNN)

A neural network is a computational system that simulates neurons of the brain. Every neural network has input, hidden, and output layers. Each layer has a structure in which multiple nodes are connected by edges. A “deep neural network” is a network in which multiple layers are used for the hidden layer. Machine learning using a deep neural network is called “deep learning.” A CNN is a type of deep neural network that has been proven to be highly efficient in image recognition. A CNN does not require predefined image features. In this study, we proposed the use of a CNN to predict the sex of patients from an FDG PET-CT image.

### Architectures

**Figure 1:**
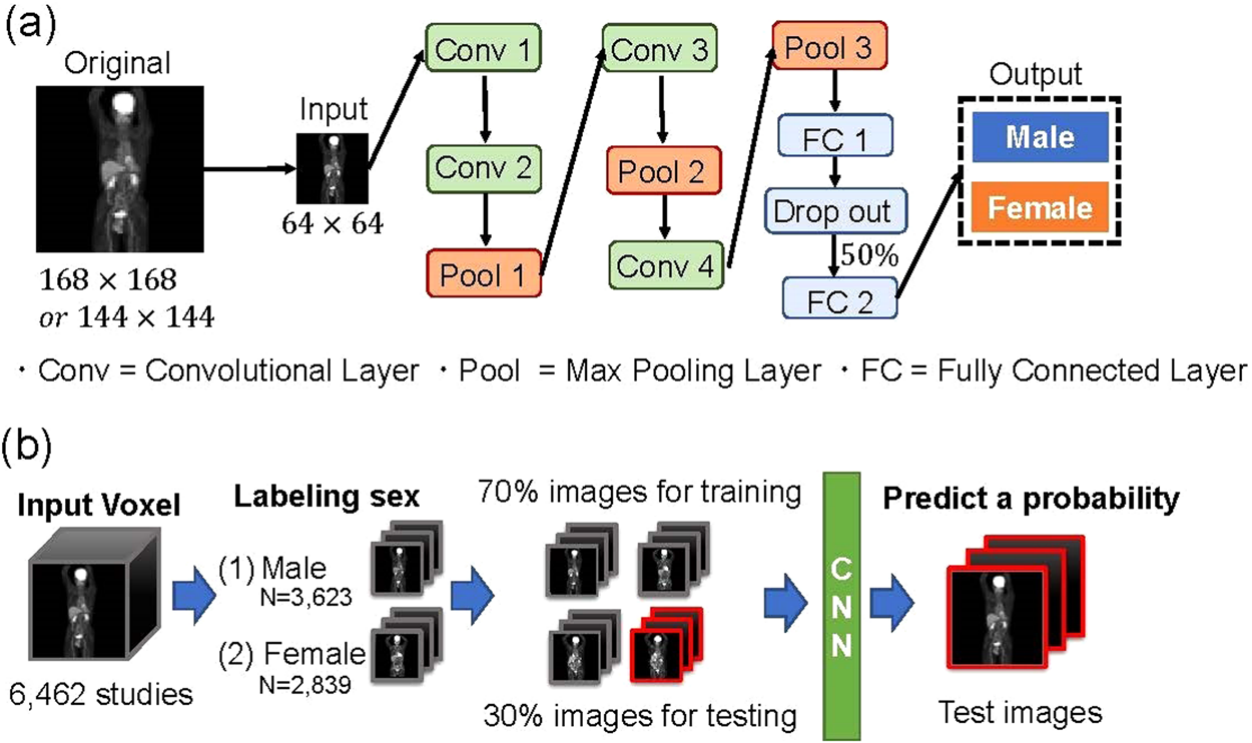
(**a**) The functional architecture of a convolutional neural network (CNN). (**b**) Training and testing process.

In this study, we designed a CNN architecture to predict patient sex from FDG PET-CT images. Here we provide details on the CNN architecture and associated techniques used in this study. The detailed architecture is shown in Fig. 1a. Each neuron in a layer is connected to the corresponding neurons in the previous layer. The architecture of the CNN used in the present study contained four convolutional layers. This network also applied a ReLU function, local response normalization, and softmax layers. The softmax function is defined as follows:

$${\rm{F}}({x}_{i})=\frac{exp({x}_{i})}{{\sum }_{j}exp({x}_{j})}$$

where *x*_*i*_ is the output of the neuron i (i = 1, 2, …, n; n being the number of neurons in the layer).

An input image is presented to the first layer, i.e., “Conv1” of Fig. 1a. The number of neurons in the first layer is equal to the number of pixels in the input gray-scaled image. There are two types of information processing that are applied iteratively: convolution and pooling. The convolution process works as a filter that extracts features from images or data in the previous layer. There are many filters in the convolution that are applied simultaneously. The parameter of the filters, which defines the feature to be extracted, is adjusted by learning algorithms. The size of the filters is smaller than that of the layer, so that the filter is repeatedly applied within a stratum. The pooling process selects the strongest activated value for a feature in a local area that is extracted in the convolution. Through pooling, even if the image is shifted slightly, the classification results are not affected, and the size of the layer is reduced by one-quarter. For the final layer, all of the neurons in one layer are connected to all of the neurons in the previous layer. The number of neurons in the final layer is equivalent to the number of class labels to be recognized. By using the softmax function, the output of the CNN can be represented as a probability. In the regression models to predict age and weight, a linear function was used for the final layer.

### Data augmentation

In this research, the number of images used for learning processes was increased 5-fold by image augmentation processing such as rotation, enlargement/reduction, parallel movement, and noise addition. Note that the original images used for the test were not subjected to such an augmentation process.

### Model training and testing

In the model training phase, we used “early stopping” and “dropout” to prevent overfitting. Early stopping is a function used to monitor the loss function of training and validation and to stop the learning before it falls into excessive learning^15–17^. Early stopping and dropout have been adopted in various machine-learning methods^18^. In the model test phase, we tested both an image-based method and a patient-based method. Each patient-based diagnosis was determined with the use of MIP images based on majority rule (19 MIP images for Scanner 1, and 36 MIP images for Scanner 2).

#### Experiment 1 (Overall)

All patients examined with the two scanners were mixed, and 4,462 (70%) randomly selected cases were extracted and learned as training data. After that, the remaining 2,000 cases (30%) were extracted and tested as test data. The process is shown in Fig. 1b. We repeated the process five times to calculate the accuracy.

#### Experiment 2 (Scanner 1 for training and Scanner 2 for testing)

We used all of the cases of Scanner 1 for training and all of the cases of Scanner 2 for testing.

#### Experiment 3 (Scanner 2 for training and Scanner 1 for testing)

Inversely, we used all of the cases of Scanner 2 for training and all of the cases of Scanner 1 for testing.

#### Experiment 4 (Masking)

**Figure 2:**
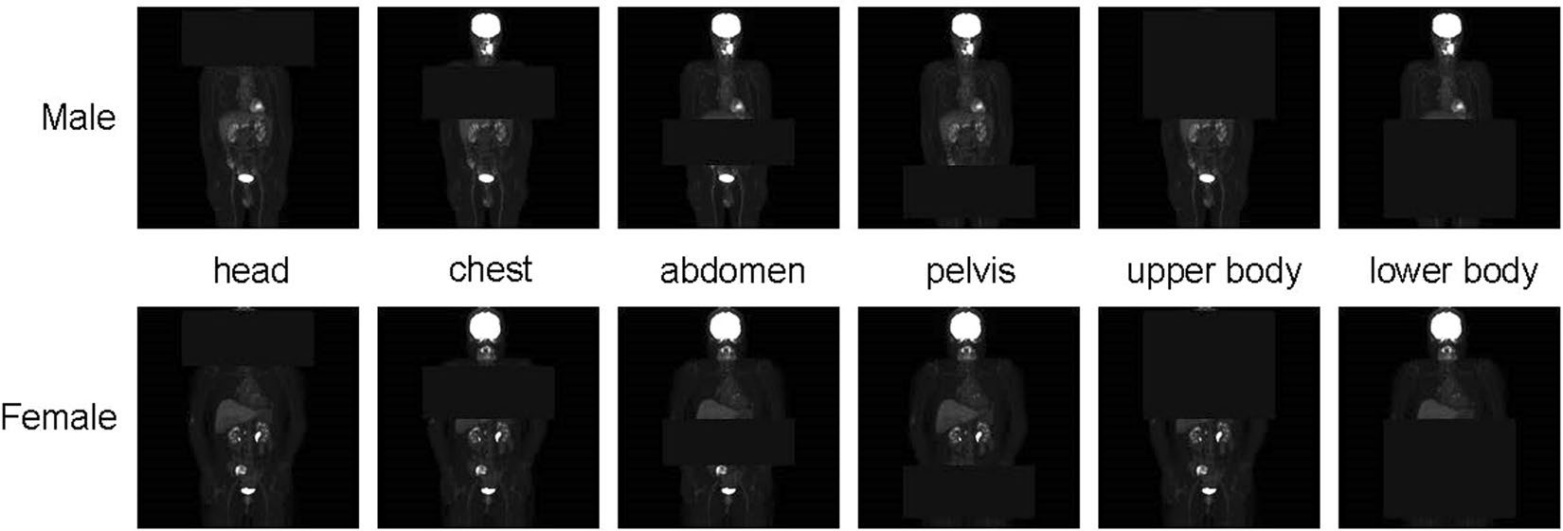
Sample mask images. The average value of the entire image was used as the pixel value of the mask set of each image. Each mask location was determined based on a typical image of a patient with average height and weight, and then applied to all the other patients’ images.

To specify the important part of the image, we conducted a “mask” experiment. As shown in Fig. 2, images (mask images) were created to cover a part of the original image. After masking, training and testing were performed under the same conditions as in Experiment 1. Six different masks were employed, respectively covering the (1) head, (2) chest, (3) abdomen, (4) pelvis, (5) upper body (=(1) +(2)), and (6) lower body (=(3) +(4)). The average value of the entire image was used as the pixel value to fill each region. Each mask location was determined based on a typical image of a patient with average height and weight, and then applied to all of the other patients’ images. The upper body was set as the head and chest, and the lower body was set as the abdomen and pelvis.

#### Experiment 5 (Grad-CAM)

We carried out additional experiments using a Grad-CAM technique, which visualizes the part activated by the neural network during diagnosis. The same image as the original image used in Experiment 4 was used as the input image.

#### Experiment 6 (ResNet)

ResNet 50 was trained and tested under the same conditions as in Experiment 1.

#### Experiment 7 (Age prediction)

A regression model to predict age by CNN was generated, trained, and tested under the same conditions as in Experiment 1.

#### Experiment 8 (Body weight prediction)

A regression model to predict body weight by CNN was generated, trained, and tested under the same conditions as in Experiment 1.

### Hardware and software environments

#### This experiment was performed under the following environment

OS, Windows 10 pro 64 bit; CPU, intel Core i7-6700K; GPU, 1x NVIDIA GeForce GTX 1080Ti 11GB; Framework, Keras 2.0.2 and TensorFlow 1.3.0; Language, Python 3.5.2; CNN, original CNN (Convolution layer, 4; Maxpooling layer, 3); Optimizer, Adam.

## Results

**Figure 3:**
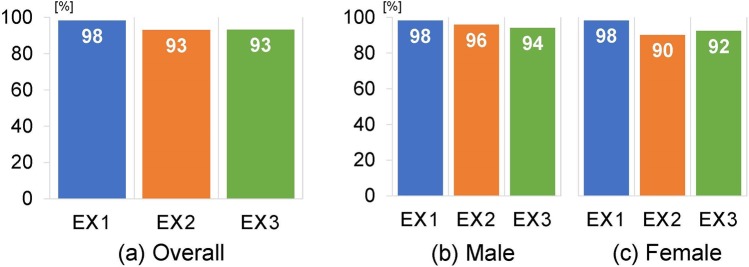
(**a**) The overall accuracy of Experiments 1–3 and the accuracy for (**b**) male and (**c**) female patients.

We retrospectively analyzed the cases of 6,462 patients (3,623 males [56%] and 2,839 females [44%]) who underwent FDG PET-CT imaging between January 2015 and August 2017 for diagnosis of various cancers at our institution. A total of 137,500 MIP images were used, and the male and female datasets consisted of 77,000 and 60,500 images, respectively. The results of Experiments 1 to 3 are summarized in Fig. 3.

### Experiment 1 (Overall)

**Figure 4:**
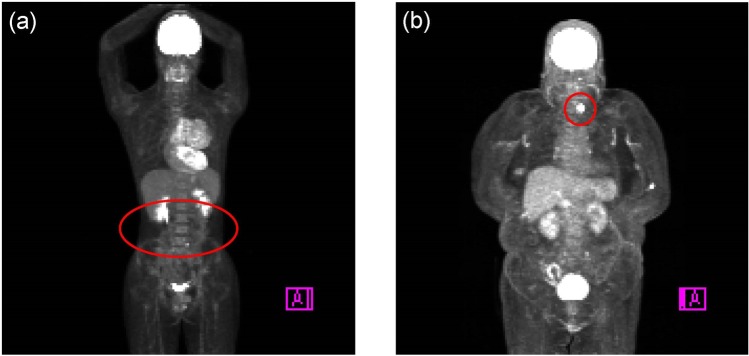
Two patients (**a**, male; **b**, female) whose sex was incorrectly predicted.

The model was trained for 5 to 10 epochs by the early stopping algorithm. The CNN process spent approx. 5 min for training each fold dataset and <10 seconds per patient for prediction. The accuracy reached 98.9 ± 0.002% for the training dataset. When images for the testing (which had not been used for the training) were given to the trained model, the accuracy was 98.2 ± 1.3% for the image-based classification. For the patient-based classification, the patient sex was predicted from MIP images based on majority rule. The overall accuracy was 99.6 ± 0.5% for the patient-based classification. The accuracy values for the identification of “male” and “female” in the image-based classification were almost the same: 98.2 ± 1.2% for male, and 98.3 ± 1.4% for female prediction. Figure 4 provides representative images for which the patient sex was incorrectly predicted.

### Experiment 2 (Scanner 1 for training and Scanner 2 for testing)

When the dataset of Scanner 1 was used for training (i.e., Scanner 2 was not used for training), a total of 5,641 cases were provided. The model was trained for 8 epochs by the early stopping algorithm. The CNN process spent 2 min for training each fold dataset and <10 seconds per patient for prediction. The accuracy reached 99.4% for the training dataset. When images of Scanner 2 for testing (which were not used in the training) were given to the learned model, the accuracy was 93.0% for the image-based classification. For the patient-based classification, the patient sex was predicted from MIP images based on the majority rule. The overall accuracy was 95.3% for the patient-based classification. The accuracy values for the male and female image-based classification were 95.9% for male and 90.2% for female.

### Experiment 3 (Scanner 2 for training and Scanner 1 for testing)

When the dataset of Scanner 2 was used for training, a total of 821 cases were provided. The model was trained for 7 epochs by the early stopping algorithm. The CNN process spent approx. 2 min for training each fold dataset and <10 seconds per patient for prediction. The accuracy reached 99.3% for the training dataset. When images of Scanner 1 for testing (which had not been used for the training) were given to the learned model, the accuracy was 93.2% for the image-based classification. For the patient-based classification, the patient sex was predicted from the MIP images based on majority rule. The overall accuracy was 94.6% for the patient-based classification. The accuracy values for the male and female image-based classification were 94.1% for male and 92.4% for female.

### Experiment 4 (Masking)

To identify the part of the image by which the CNN predicted sex, we performed masking experiments. The results are summarized in Table 1. When the lower body (especially the pelvic area) was masked, the accuracy was significantly degraded. Female patients were more frequently misidentified. When the pelvic area was masked, the accuracies for male and female patients were approx. 86% and approx. 57%, respectively. When other body parts were masked, the accuracy was less degraded.

**Table 1:** Results of the mask experiment.

Mask location	Male	Female
	Accuracy	Accuracy
No mask	95%	94%
Head	94%	98%
Chest	99%	89%
Abdomen	89%	98%
Pelvis	86%	57%
Upper body	97%	91%
Lower body	80%	61%

### Experiment 5 (Grad-CAM)

**Figure 5:**
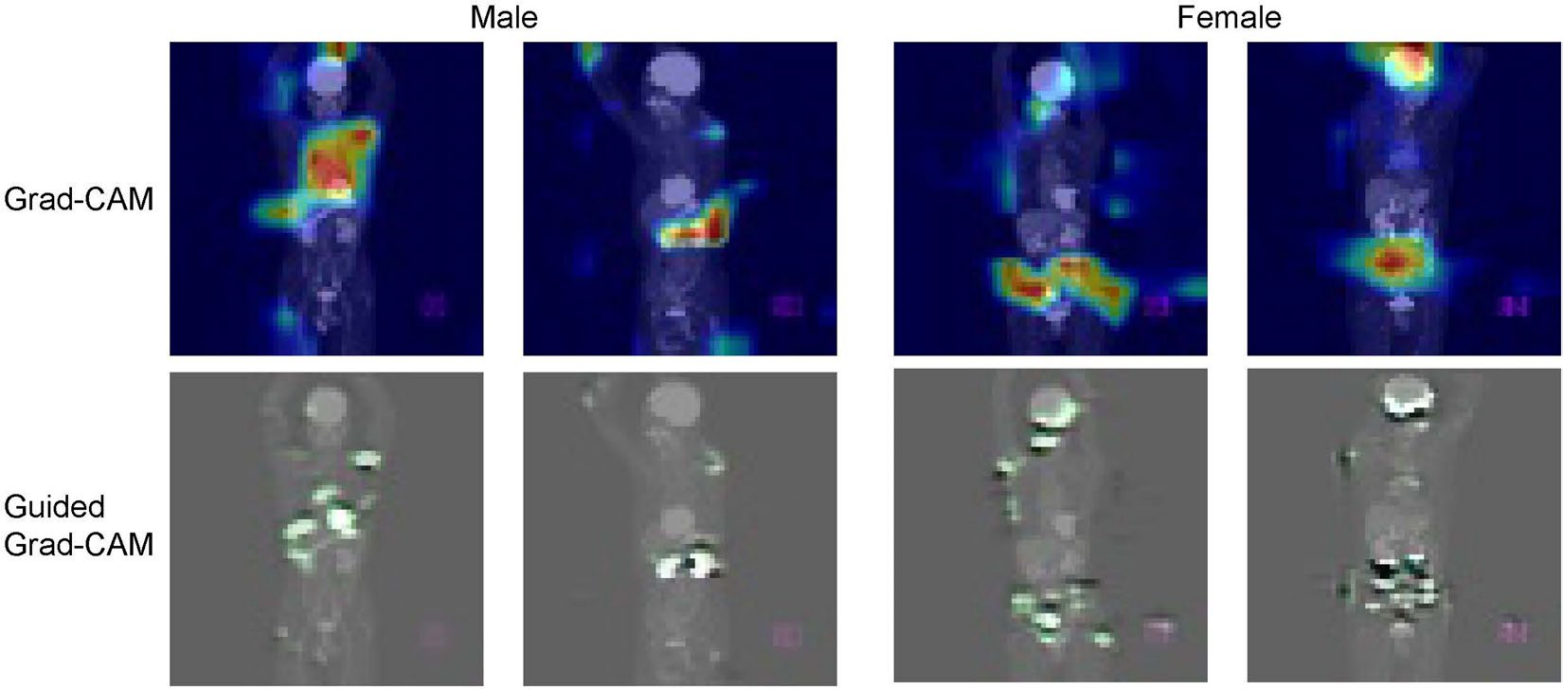
Typical examples of Grad-CAM. The areas on which the neural network focused are highlighted. The chest and abdominal regions are typically highlighted for male and female patients, respectively.

We further employed Grad-CAM to identify the part of the image the CNN paid attention to. Typical examples are shown in Fig. 5. For most cases, we observed that the chest of men and the pelvic region of women were highlighted.

### Experiment 6 (ResNet)

To test a more complicated neural network, we employed ResNet. The model was trained for 25 epochs. The CNN process spent approx. 8 hours on the training dataset and <10 seconds per patient for prediction. The accuracy reached 99.8% for the training dataset. When images for the testing (which had not been used for the training) were given to the trained model, the accuracy was 99.8% for the image-based classification. For the patient-based classification, the patient sex was predicted from MIP images based on the majority rule. The overall accuracy was 99.9% for the patient-based classification. The accuracies for the identification of male and female in the image-based classification were 99.7% and 99.9%, respectively.

### Experiment 7 (Age prediction)

**Figure 6:**
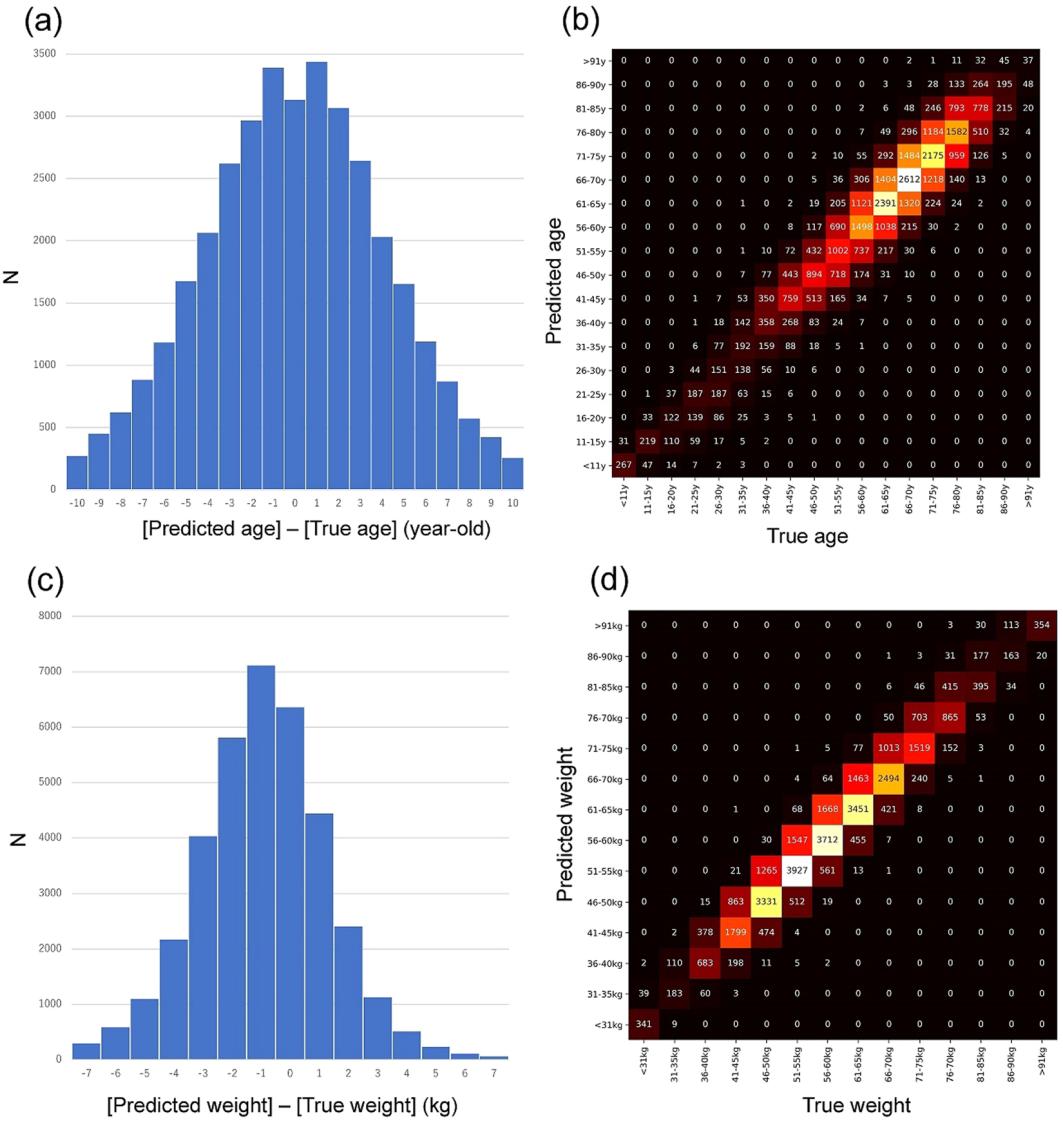
(**a**) Difference between predicted and true ages. (**b**) Confusion matrix of predicted and true ages. (**c**) Difference between predicted and true weights. (**b**) Confusion matrix of predicted and true weights.

Age was predicted using a regression model. The model was trained for 50 epochs. When images for the testing (which were not used for the training) were given to the trained model, 83.2% of patients were accurately predicted with absolute error being smaller than 5 years. Also, 97% were predicted within ± 10 years. Figure 6a,b are mixing matrices with age being divided into 18 steps (0‒10, 11‒15…86‒90, >91 years old).

### Experiment 8 (Body weight prediction)

Body weight was predicted using a regression model as well. The model was trained for 50 epochs. When images for the testing (which had not been used for the training) were given to the trained model, 96.1% of patients were accurately predicted, with the absolute error being smaller than 5 kg. Also, 98% were predicted within ± 6 kg. Figures 4d and 6c are mixing matrices with body weight being divided into 14 steps (0‒30, 31‒35, 36‒40… 86‒90, >91 kg).

## Discussion

In this research using a CNN, approx. 98.2% of MIP images of FDG PET were correctly categorized by sex, and the sexes of approx. 99.6% of the patients were correctly categorized. These data suggest that a CNN can predict the patient sex from MIP images of FDG PET. An additional alert system that reveals patient sex mismatches and informs medical staff would help prevent misidentification accidents.

To further reveal the characteristics of the current CNN, we conducted two additional experiments (Experiments 2 and 3) to evaluate scanner effects. In both experiments, images from different scanners were used for training and testing, respectively. The accuracy was slightly lower in Experiments 2 and 3 compared to that in Experiment 1. These results suggest that scanner-dependent image quality (e.g., spatial resolution, noise level, matrix size), in addition to the different numbers of patients scanned by each scanner, may affect the performance of the CNN. The accuracy was sufficiently high when two types of PET scanners were used in combination (Experiment 1). These results indicate that the CNN developed in this study could acquire versatility by learning versatile images, suggesting that it is important to use as many scanners as possible to commercialize a CNN for practical applications.

On what part of the image did the CNN focus to distinguish between males and females? To address this question, we conducted additional simulations, Experiments 4 and 5. In Experiment 4, comparing image masks of several regions, we found that the accuracy was lowest when the pelvic area was masked, indicating—not surprisingly—that the most important features to discriminate patients by sex lie in the pelvic area. This result is intuitive, of course, although various other parts of the body also show sex differences. For example, brain metabolism may differ by sex^19^, though in our model the CNN was unable to use this information, since the images of the brain were all saturated—i.e., blackened—due to the SUV window setting of 0–10. The breasts/chest may be an important locus of sex information, but there is a large variation in the degree of FDG accumulation in this region among women, depending on age and estrogenic status. In Experiment 5, Grad-CAM typically highlighted the chest region for men and the pelvic region for women. These results, together with the results of Experiment 4, suggested that both the chest and pelvic regions can have important information for identifying men. In contrast, for women, the pelvic region is important, but the chest region is less important, possibly because women have more inter-individual variability in the chest region (e.g., size and metabolism of the breasts) than in the pelvic region.

We investigated some cases in which the patient’s sex was incorrectly predicted in Experiment 1. One patient (Fig. 4a) was male but so slender that he might have been confused for a female. Another patient (Fig. 4b) was female; she was relatively obese, and she had head-and-neck cancer, which is a male-dominant disease. These factors might have led to the misprediction by the CNN.

Experiment 6 was carried out to determine whether the accuracy might be improved by using a more complicated network such as GoogLeNet^20^ or ResNet^21^. The results showed that these alternative networks took more time to train compared with the simple network used in Experiments 1 to 3, but they required roughly the same amount of time as our network to make their diagnoses. Thus, while the training process required approximately 100 times more time in Experiment 6 than in Experiment 1, the predictions in Experiment 1 and Experiment 6 both took less than 10 seconds per patient. The accuracy improved from 98.2% to 99.8%, suggesting that an improvement in learning accuracy can be expected by applying a complex network.

We considered that patient misidentification accidents could be further prevented if not only patient sex, but also patient age and weight could be predicted from images and compared against known data. In Experiments 7 and 8, we tested the ability of our regression model to predict age and weight. The results showed that both age and body weight were appropriately estimated. Although the system was not always able to deliver precise predictions within 1 year of age or 1 kg of weight, the prediction system using the combination of sex, age, and body weight would contribute to a reduction in misidentification accidents.

**Figure 7:**
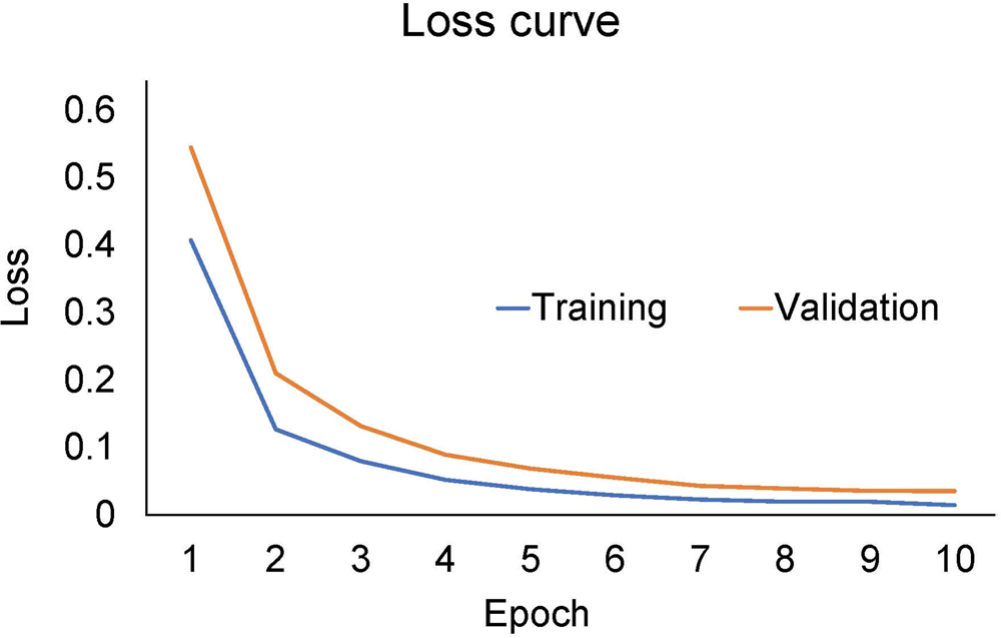
Loss curves of training and validation in this study. The training was completed at 10 epochs due to early stopping, and both the training and evaluation gradually declined.

There are two problems associated with machine learning or deep learning: underfitting and overfitting. Both can be represented by a loss curve against epochs^15^. When underfitting occurs, the loss curve continues to decline for both training and validation. In overfitting, the loss curve of training approaches 100%, whereas the loss curve of validation moves away from 100%. We detected no evidence of underfitting or overfitting in the present study, as the loss curves at training and validation (Fig. 7) shifted in the same way.

The computational complexity becomes enormous when a CNN directly learns with 3D images^22–24^. Another approach is to let a CNN learn slice images instead of MIP images. However, there are many slices that do not contain sex information. In contrast, MIP seems to be advantageous for a CNN because all MIP images may contain sex information somewhere in the image. For example, in a typical male case, of all 545 slices from the head to the thigh, only 13 (2%) slices covered the testes, although all the MIP images showed the testes. In a typical female case, of all 478 slices from the head to the thigh, only 36 (8%) slices covered the breasts, although all the MIP images showed the breasts. MIP images can also be directly generated from PET, in contrast to CT, for which bed removal is necessary before MIP generation. In addition, if a single MIP image from a single angle is fed to the network, the patient sex cannot always be predicted accurately. In the current study, however, MIP images generated from various angles were given to the neural network. This might have improved diagnostic accuracy.

Deep learning has the potential to automate various tasks. Indeed, this technology has been used not only for diagnosis but also for image generation, and especially for image quality improvement and reduction of the radiation dose^25–27^. Combining our current network and others will contribute to safe medical imaging by reducing the misidentification incidents and radiation exposure and by preventing misdiagnosis.

This study has some limitations. First, because our model was generated for whole-body images, spot images such as pelvic areas could not be recognized. Further studies will be needed, such as investigations of the potential improvement to the training data by cropping different areas. Second, while we investigated the use of images from 2 different scanners, there are many more scanners currently used in the world. In order to cope with various multicenter scanners, training dataset should have images of as many scanners as possible. Finally, it is not yet known how the accuracy of a CNN system changes if a tumor exists in the patient’s pelvis.

## Conclusion

Our findings indicate that the CNN-based sex prediction system successfully classified patients by sex, age and body weight categories. Such a system may be useful to prevent patient misidentification accidents in clinical settings.
